# Changes in cerebral metabolites in obstructive sleep apnea: a systemic review and meta-analysis

**DOI:** 10.1038/srep28712

**Published:** 2016-06-28

**Authors:** Yunyan Xia, Yiqun Fu, Huajun Xu, Jian Guan, Hongliang Yi, Shankai Yin

**Affiliations:** 1 Department of Otolaryngology Head and Neck Surgery & Center of Sleep Medicine, Shanghai Jiao Tong University Affiliated Sixth People’s Hospital, Yishan Road 600, Shanghai, 200233, China.; 2 Otolaryngological Institute of Shanghai Jiao Tong University, Yishan Road 600, Shanghai, 200233, China.; 3 Clinical Research Center, Shanghai Jiao Tong University School of Medicine, South Chongqing Road 225, Shanghai, 200020, China.

## Abstract

Cognitive impairment is associated with changes in cerebral metabolites in patients with obstructive sleep apnea (OSA). Several studies have used magnetic resonance spectroscopy (MRS) to detect variations in cerebral metabolites; however, the results have been inconsistent. This meta-analysis summarizes the differences in cerebral metabolites between patients with OSA and controls. Two electronic databases, PubMed and Embase, were searched for articles (published before March 31, 2016) describing studies that used MRS to evaluate the cerebral metabolite changes. The overall effects were measured using the weighted mean difference with a 95% confidence interval. Subgroup analysis and sensitivity analysis were used to explore the sources of between-study heterogeneity and the stability of the results. Publication bias was also evaluated. Thirteen studies were ultimately included. In the hippocampus, the N-acetylaspartate (NAA)/creatine ratio was lower in patients with OSA. In the frontal lobe, only the NAA/choline ratio was lower in patients with OSA. Cerebral metabolites are significantly altered in the hippocampus in patients with OSA. Further clinical studies are needed to explore the underlying mechanisms between OSA and the changes in cerebral metabolites in the brain.

Obstructive sleep apnea (OSA) is one of the most common sleep disturbances, affecting 4% to 9% of the adult population^1^,^2^. It is characterized by repetitive complete (apnea) or partial (hypopnea) obstruction of the upper airway during sleep. The pathophysiology of OSA includes oxygen desaturation, micro-arousals, and abnormal ventilation and sleep architecture^3^. OSA can increase the risk of cardiovascular disease^4^, stroke^5^, metabolic disease^6^, excessive daytime sleepiness^7^, work-place errors, traffic accidents^8^, and death^9^, resulting in significant economic burden. Therefore, it is an important health problem that should not be overlooked^10^. Altered cognitive functions have recently been reported in patients with OSA, including inattention, poor memory, and general intellectual and executive dysfunctions^11^,^12^. These cognitive impairments could affect the quality of life of patients with OSA.

Structural-metabolic changes in some regions of the brain are responsible for the occurrence of cognitive impairment^13^. Magnetic resonance spectroscopy (MRS) is a noninvasive method commonly used to evaluate local changes in metabolites, such as N-acetylaspartate (NAA), choline (Cho), creatine (Cr), glutamate and glutamine (Glx), and myo-inositol (mI). NAA is a marker of neuronal integrity, while Cho is associated with membrane metabolism^14^. Cr reflects energy metabolism, Glx regulates neurotransmitter activity, and mI is considered a glial cell marker^14^. Measurements of these metabolites serve as an indicator of cerebral impairment, including neuronal loss, axonal injury, and gliosis.

Many small-scale studies have sought to determine whether the cerebral metabolites in brain regions differ between patients with OSA and controls to determine the pathophysiology of cognitive impairment in patients with OSA. However, the results have been inconsistent. To the best of our knowledge, no systematic review or meta-analysis has evaluated the changes in cerebral metabolites in patients with OSA. Therefore, we examined this issue in the present study.

## Results

### Search results

**Figure 1:**
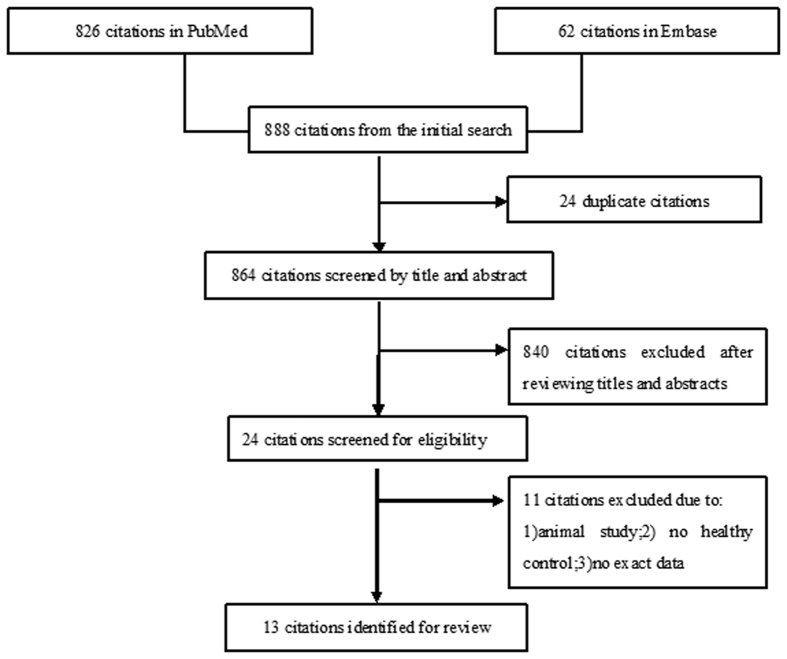
Flow diagram of selection procedure in this meta-analysis showing the number of records retrieved and the number of studies included.

The initial search retrieved 888 references from PubMed (n = 826) and Embase (n = 62). Of these 888 citations, 24 papers were initially removed due to duplication. A further 840 records were removed after screening by titles and abstracts. For the remaining 24 papers, we carefully reviewed the full text and excluded an additional 11 articles due to the following reasons: (1) animal study, (2) control group was not healthy, or (3) no exactable data^15^,^16^,^17^,^18^,^19^,^20^,^21^,^22^,^23^,^24^,^25^. Ultimately, 13 articles (269 cases and 233 controls) evaluating changes in cerebral metabolites in OSA were included. The flow diagram of the search procedure is shown in Fig. 1.

### Study characteristics

The 13 articles were published between February 1997 and February 2016: three were studies conducted in America^26^,^27^,^28^; two each were studies conducted in Italy^29^,^30^, Australia^31^,^32^, and Turkey^33^,^34^; and the remainder were from Greece^35^, Egypt^36^, Japan^37^, and India^38^. Most of the subjects were middle-aged, while only one study focused on pediatric OSA^26^. The mean Epworth Sleep Scale (ESS) score ranged from 8.2 to 11.7, and the mean apnea-hypopnea index (AHI) was 19.0 to 71.5 events per hour in patients with OSA. The percentage of males ranged from 64% to 100% and mean body mass index (BMI) was 26.4 to 47.3 kg/m^2^ in the OSA groups. According to the Newcastle–Ottawa Scale (NOS) guidelines, three studies scored 6 points, eight scored 5 points, and two scored 4 points. This information is summarized in Table 1.

### Main results

Five studies^26^,^31^,^32^,^33^,^34^ evaluated the changes in the NAA/Cr ratio in the hippocampus [weighted mean difference (WMD), −0.08; 95% confidence interval (CI), −0.14 to −0.02; *p* < 0.01). There was no evidence of significant between-study heterogeneity (I^2^ = 0%, *p* = 0.52). Four studies^26^,^32^,^33^,^34^ evaluated changes in the NAA/Cho ratio in the hippocampus (WMD, −0.12; 95% CI, −0.36 to 0.11; *p* = 0.30); however, there was significant between-study heterogeneity (I^2^ = 77%, *p* < 0.01). Four studies^26^,^32^,^33^,^34^ evaluated changes in the Cho/Cr ratio in the hippocampus (WMD, −0.01; 95% CI, −0.10 to 0.08; *p* = 0.78) and showed significant heterogeneity (I^2^ = 63%, *p* = 0.05). Six studies^26^,^29^,^32^,^33^,^35^,^36^ evaluated changes in the NAA/Cr ratio in the frontal lobes (WMD, −0.36; 95% CI, −0.77 to 0.06; *p* = 0.09), with heterogeneity (I^2^ = 97%, *p* < 0.01). Four studies^26^,^32^,^33^,^35^ evaluated changes in the NAA/Cho ratio in the frontal lobes (WMD, −0.32; 95% CI, −0.64 to −0.01; *p* = 0.05), with heterogeneity (I^2^ = 86%, *p* < 0.01). Five studies^26^,^29^,^32^,^33^,^35^ evaluated changes in the Cho/Cr ratio in the frontal lobes (WMD, −0.02; 95% CI, −0.14 to 0.09; *p* = 0.68), with heterogeneity (I^2^ = 94%, *p* < 0.01). This information is shown in Table 2. Because the units of measurement for NAA, Cr, and Cho varied among the included studies, we were unable to pool the results; we have presented the data in Supplementary Table 1.

### Subgroup analyses

#### Changes in cerebral metabolites in the frontal white matter

Three studies^33^,^35^,^36^ evaluated changes in the NAA/Cr ratio (WMD, −0.51; 95% CI, −1.11 to 0.09; *p* = 0.09), and three studies^33^,^35^,^36^ evaluated changes in the Cho/Cr ratio (WMD, −0.06; 95% CI, −0.31 to 0.19; *p* = 0.64). All showed significant between-study heterogeneity (Table 3).

#### Changes in cerebral metabolites in the frontal cortex

Three studies^26^,^33^,^35^ evaluated changes in the NAA/Cr ratio (WMD, −0.13; 95% CI, −0.39 to 0.13; *p* = 0.32), and the same three evaluated changes in the NAA/Cho ratio (WMD, −0.43; 95% CI, −1.07 to 0.20; *p* = 0.18); there was significant between-study heterogeneity (see Table 3). These studies^26^,^33^,^35^ also evaluated changes in the Cho/Cr ratio (WMD, −0.01; 95% CI, −0.04 to 0.03; *p* = 0.80), without heterogeneity (I^2^ = 20%, *p* = 0.29).

### Sensitivity analysis

A sensitivity analysis was performed by sequentially omitting one study at a time to examine the influence of each study. If significant between-study heterogeneity existed, a sensitivity analysis was performed to assess the stability of the results.

In the hippocampus, after excluding the study by Halbower *et al*.^26^, the Cho/Cr ratio significantly decreased in patients with OSA without heterogeneity (I^2^ = 0%, *p* = 0.75); the significant heterogeneity of the NAA/Cho ratio also disappeared (I^2^ = 0%, *p* = 0.49). Excluding any single study did not influence the pooled results for the NAA/Cho ratio (Supplementary Table 2).

In the frontal lobe, no single study influenced the pooled results for the NAA/Cr and Cho/Cr ratios. The NAA/Cho ratio, after excluding the study by Alchanatis *et al*.^35^, significantly decreased in patients with OSA without significant heterogeneity (I^2^ = 0%, *p* = 0.49). However, after excluding the studies by Algin^33^, Halbower^26^, or O’Donoghue *et al*.^32^, there was no significant difference between patients with OSA and controls, but significant heterogeneity was present (Supplementary Table 2).

### Publication bias

To assess publication bias among the included cross-sectional studies, Begg’s rank correlation test and the Egger linear regression test were performed. The *p*-values of these two tests were >0.05, indicating that there was no significant publication bias in evaluating each cerebral metabolite in our meta-analysis.

## Discussion

Emotional-cognitive changes and daytime sleepiness are common symptoms of OSA. Structural-metabolic changes in some regions of the brain are responsible for these symptoms^13^. Neuroimaging studies of patients with OSA might help clarify the biological mechanisms underlying the onset and duration of the disease^13^. They can also help to identify neural abnormalities associated with brain function in patients with OSA. MRS can provide data on the metabolic status of the tissue by quantifying the cellular metabolites and probing their variation during the disease processes; it can also detect microstructural changes in the brain that are not visible in conventional cranial MRI. Therefore, we combined all MRS studies of cerebral metabolites to achieve a consensus.

Previous studies using MRS have revealed that cerebral metabolites vary in regions of the brain in patients with OSA, including the hippocampus, thalamus, cerebral cortex, white matter, insular cortex, brain stem, cortex, and the frontal, temporal, and occipital lobes. In this meta-analysis, the pooled results showed that OSA was associated with a low NAA/Cr ratio in the hippocampus with no significant between-study heterogeneity and a low Cho/Cr ratio in the hippocampus after excluding the study by Halbower *et al*.^26^. In the frontal lobe, OSA was associated with a low NAA/Cho ratio, but this negative relationship was not stable because of varying results in sensitivity analysis. A decrease in NAA is notable in diseases with neuro-axonal loss or dysfunction, such as Alzheimer disease^39^ and cerebral ischemia^40^, while an increase in the NAA signal was only seen in Canavan disease^41^. Cr has neuroprotective properties^42^ and enhances neurocognitive abilities^43^. The levels of hippocampal Cr respond to hippocampal exercise^44^, which increase in hypometabolic states and decrease in hypermetabolic states. A decrease in the Cr signal in the hippocampus indicates the loss of hippocampal function in patients with OSA. An elevated Cho level reflects increased membrane turnover or increased cellular density and has been reported in cases of active demyelination, brain tumors, and glial proliferation^14^,^45^,^46^, whereas lower Cho levels have been reported in mitochondrial, hypomyelination, metabolic, and liver diseases^14^,^45^; Grave’s disease^47^; Lewy body dementia^48^; and chronic obstructive pulmonary disease^49^. Increases or decreases in Cho may represent different stages in the evolution of the same pathological process, with increased lipid turnover initially and subsequent decreased turnover and possible apoptosis. Thus, changes in cerebral metabolites in the frontal lobe and hippocampus may demonstrate injury to these regions.

The frontal lobe, especially the prefrontal cortex and the prefrontal-subcortical brain circuits^50^, is associated with cognitive and executive function. Therefore, lesions in the prefrontal cortex and the prefrontal-subcortical brain circuits can cause cognitive impairment and executive dysfunction, as can lesions in the anterior white matter, by interrupting the prefrontal-subcortical circuits^50^,^51^,^52^. Previous studies have reported that OSA could induce chemical and structural cellular injury and affect the prefrontal cortex^53^,^54^,^55^. In this meta-analysis, cerebral metabolite changes associated with OSA in the frontal lobe might reflect injury to these regions, leading to mild cognitive impairment such as inattention, poor memory, and poor executive function. In addition to the frontal lobes, changes in the cerebral metabolites were also seen in the hippocampal area, which is metabolically active and highly susceptible to hypoxic insult. This brain region is closely associated with learning skills, memory, and advanced mental activity, which might explain some of the cognitive deficits in patients with OSA.

**Figure 2:**
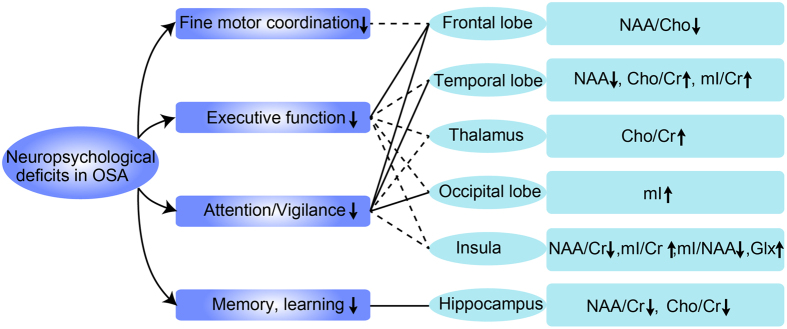
Neuropsychological deficits and cerebral metabolite changes in respective regions in OSA. Abbreviation: OSA, obstructive sleep apnea; NAA, N-acetylaspartate; Cho, choline; Cr, creatine; mI, myo-inosito.

To clearly understand the variations in the metabolites in different cerebral regions, we have summarized all of the results from the different studies in Fig. 2. First, in the thalamus, the Cho/Cr ratio increased in patients with OSA, while the NAA/Cr and NAA/Cho ratio did not. Second, in the parietal-occipital white matter, the levels of NAA, Cho, Cr, and mI and the ratios of NAA/Cr, NAA/Cho, Cho/Cr, and mI/Cr were unchanged. Third, in the brain stem, the ratios of NAA/Cho, NAA/Cr, and Cho/Cr were also unchanged. Fourth, in the temporal lobe, the level of NAA decreased; the ratios of Cho/Cr and mI/Cr increased; and the Cr, Cho, Glx, and mI levels and the NAA/Cr ratio were unchanged. Fifth, the NAA/Cr and mI/NAA ratios decreased and the mI/Cr ratio and Glx level increased in the insular cortex, while the Cho/Cr ratio was unchanged. Finally, in the occipital lobe, only the mI level increased, while the NAA, Cr, Cho, and Glx levels were unchanged. We conclude that the neuropsychological deficits in patients with OSA, including impaired executive dysfunction, fine motor coordination, memory and learning skills, and attention/vigilance, may be related to the metabolite changes in specific cerebral regions.

To the best of our knowledge, this is the first meta-analysis to investigate the changes in cerebral metabolites in patients with OSA. Despite the interesting findings mentioned above, some limitations should be addressed. First, no firm conclusions could be drawn because of the relatively small sample size and varying results in the sensitivity analysis. Second, we included only English-language studies, which might have resulted in selection bias. Third, although we stratified the changes in cerebral metabolites according to brain region, there was significant between-study heterogeneity. This may have been due in part to the small number of participants and unadjusted confounding factors.

In conclusion, cerebral metabolites are significantly altered in the hippocampus in patients with OSA as evidenced by the low NAA/Cr ratios in the hippocampus and the low NAA/Cho ratio in the frontal lobe. These abnormalities of cerebral metabolites might be a pivotal bridge connecting cognitive deficits and OSA.

## Materials and Methods

This systemic review and meta-analysis was performed according to the Preferred Reporting Items for Systematic Reviews and Meta-Analyses (PRISMA) statement^56^. Ethical approval was not necessary for this meta-analysis because each included study had already received ethical approval.

### Search strategy

A systematic search was performed using two electronic databases, PubMed and Embase, and the final search was conducted on March 31, 2016. The search terms were as follows: (obstructive sleep apnea) OR (sleep apnea syndromes) OR (OSA) OR (sleep apnea) combined with (magnetic resonance spectroscopy) OR (MRS) OR (magnetic resonance imaging). Additional reports were added when discovered by citation tracking. This search was performed separately by Drs. Xia and Fu.

### Inclusion and exclusion criteria

We included studies that satisfied the following inclusion criteria: (1) OSA was confirmed by polysomnography, (2) MRS was used to detect cerebral metabolites in patients with OSA, (3) the results were published in English, and (4) there were sufficient data to extract and estimate the WMD and the 95% CI for differences in cerebral metabolites between patients with OSA and controls. We excluded studies if they were nonclinical studies or had no control group.

### Data extraction

All studies that met the criteria were retrieved and the required information was extracted by both reviewers. The details extracted from each study included the first author, publication year, number of enrolled subjects, percentage of males, BMI, AHI, and ESS in subjects and controls. Details on metabolites were also extracted, such as the NAA, Cho, Cr, Glx, and mI levels and the NAA/Cr, Cho/Cr, NAA/Cho, mI/Cr, and Glx/Cr ratios. Disagreements between reviewers were resolved by consensus through a group discussion.

### Assessment of study quality

The NOS score was used to assess the quality of the included studies^26^,^27^,^28^,^29^,^30^,^31^,^32^,^33^,^34^,^35^,^36^,^37^,^38^. The NOS score includes three categories (selection, comparability, and exposure) with a total of eight items^57^. For each category, a study was given a maximum of two points. Studies that were awarded 0–3, 4–6, or 7–9 points were recognized as low-, intermediate-, and high-quality studies, respectively.

### Statistical analysis

RevMan (ver. 5.3, Cochrane Collaboration, Oxford, UK) and Stata (ver. 12.0, StataCorp, College Station, TX, USA) were used for the statistical analyses. The pooled estimates of the WMD and 95% CI for continuous data were calculated using the generic inverse variance method according to Cochrane Handbook for Systematic Reviews of Interventions Version 5.1.0. The heterogeneity across the included studies was evaluated using the Q-test and I^2^ statistic^58^. A *p*-value of >0.1 indicated that there was no heterogeneity. If between-study heterogeneity existed, the DerSimonian and Laird random-effects model was applied; otherwise, a Mantel–Haenszel fixed-effects model was used. Subgroup analysis was performed to explore the source of the heterogeneity. Sensitivity analysis was performed to assess the stability of the results. Begg’s and Egger’s tests were used to evaluate publication bias^59^,^60^.

## Additional Information

**How to cite this article**: Xia, Y. *et al*. Changes in cerebral metabolites in obstructive sleep apnea: a systemic review and meta-analysis. *Sci. Rep.* **6**, 28712; doi: 10.1038/srep28712 (2016).

## Supplementary Material

Supplementary Table 1

Supplementary Table 2

## Figures and Tables

**Table 1:** Study characteristics.

		OSA						Control						
Study	Country	No.	Age	Male (%)	BMI	AHI	ESS	No.	Age	Male (%)	BMI	AHI	ESS	NOS
Kamba *et al*.^37^	Japan	23	48.5 ± 12.9	82.6	(–)	(–)	(–)	15	45.7 ± 17.6	46.7	(–)	(–)	(–)	5
Alchanatis *et al*.^35^	Greece	22	49 ± 9.7	100	(–)	70.6 ± 19.4	8.2 ± 3.3	10	42.9 ± 10.5	100	(–)	3.4 ± 1.5	(–)	6
Alchanatis *et al*.^35^	Greece	14	48 ± 10.1	100	(–)	70.1 ± 19.8	8.4 ± 2.5	10	42.9 ± 10.5	100	(–)	3.4 ± 1.5	(–)	6
Bartlett *et al*.^31^	Australia	8	47.7	100	30.8	(–)	(–)	5	51.8	(–)	25.0	(–)	(–)	5
Halbower *et al*.^26^	America	6	11.5 ± 3.5	(–)	30.0 ± 6.2	37.9 ± 8.0	(–)	6	11.0 ± 2.3	(–)	20.9 ± 4.9	0.4 ± 0.2	(–)	5
Halbower *et al*.^26^	America	**7**	11.0 ± 3.1	(–)	29.4 ± 5.8	19.0 ± 9.9	(–)	6	11.0 ± 2.3	(–)	21.0 ± 4.8	0.2 ± 0.3	(–)	5
Tonon *et al*.^30^	Italy	14	48 ± 7	100	32.3 ± 4.6	(–)	13 ± 2	10	(–)	100	25 ± 2	(–)	(–)	5
Sarchielli *et al*.^29^	Italy	20	52.7 ± 11.0	65	26.4 ± 2.1	16.7 ± 15.0	11.7 ± 1.7	20	51.4 ± 13.2	70	22.1 ± 3.4	2.7 ± 0.3	4.4 ± 0.8	5
Sharma *et al*.^38^	India	18	48.0 ± 8.8	66.7	30.0 ± 5.5	46.7 ± 29.8	12 ± 5.6	32	39.9 ± 10.2	65.6	27.2 ± 5.9	0.9 ± 1.3	5.5 ± 4.1	5
Algin *et al*.^33^	Turkey	24	52	95.8	(–)	56 ± 8	(–)	9	48	66.7	(–)	<5	(–)	5
O’Donoghue *et al*.^32^	Australia	30	45.2 ± 9.6	100	33.3 ± 4.6	71.5 ± 16.2	12.9 ± 4.3	23	41.3 ± 9.3	100	25.6 ± 2.9	5.3 ± 3.9	5.2 ± 3.4	6
Gharraf *et al*.^36^	Egypt	15	48.1 ± 6.9	73.3	47.3 ± 13.5	65.5 ± 20.3	17.7 ± 2.5	10	49.1 ± 7.6	70	26.2 ± 2.1	2.7 ± 1.2	3.4 ± 1.2	6
Kizilgoz *et al*.^34^	Turkey	20	47.7 ± 11.6	75	(–)	29.3 ± 21	(–)	5	43.8 ± 2.6	20	(–)	4 ± 0.88	(–)	4
Yadav *et al*.^28^	America	36	48 ± 9.3	77.8	30.6 ± 5.9	30.6 ± 5.9	9.9 ± 4.9	53	46.8 ± 8.1	60.4	24.7 ± 3.8	(–)	5.7 ± 3.3	5
Macey *et al*.^27^	America	14	47.5 ± 10.5	64.2	(–)	29.5 ± 15.6	(–)	22	47.5 ± 10.1	50	(–)	(–)	(–)	4

Abbreviations: OSA, Obstructive sleep apnea; No., number of enrolled subjects; Male (%), percentage of male subjects; AHI, Apnea/Hypopnea Index; BMI, Body Mass Index; ESS, Epworth Sleepiness Score; NOS, Newcastle–Ottawa Scale.

**Table 2:** Main results for metabolites in different brain regions.

Study	OSA	Control	Weight%	>MD [95%CI]	*p*	I^2^%	*p*	*P*_Begg_	*P*_Egger_
NAA/Cr in hippocampus									
Algin *et al*.^33^	1.16 ± 0.1	1.24 ± 0.1	61.0	−0.08 [−0.16, −0.00]					
Bartlett *et al*.^31^	1.73 ± 1.46	1.25 ± 0.56	0.3	0.48 [−0.64, 0.60]					
Halbower *et al*.^26^	1.22 ± 0.38	1.21 ± 0.23	2.8	0.01 [−0.35, 0.37]					
Kizilgoz *et al*.^34^	2.00 ± 0.95	2.40 ± 0.53	1.8	−0.40 [−0.84, 0.04]					
O’Donoghue *et al*.^32^	1.58 ± 0.23	1.66 ± 0.15	34.0	−0.08 [−0.18, 0.02]					
Pooled			100	−0.08 [−0.14, −0.02]	0.52	0	<0.01	0.81	0.28
NAA/Cho in hippocampus									
Algin *et al*.^33^	1.07 ± 0.1	1.11 ± 0.2	32.0	−0.04 [−0.18, 0.10]					
Halbower *et al*.^26^	0.91 ± 0.05	1.29 ± 0.21	30.1	−0.38 [−0.55, −0.21]					
Kizilgoz *et al*.^34^	2.06 ± 0.99	2.25 ± 0.67	13.1	−0.19 [−0.71, 0.33]					
O’Donoghue *et al*.^32^	4.15 ± 0.52	4.04 ± 0.45	24.9	0.11 [−0.15, 0.37]					
Pooled			100	−0.12 [−0.36, 0.11]	<0.01	77	0.30	0.09	0.01
Cho/Cr in hippocampus									
Algin *et al*.^33^	1.09 ± 0.1	1.15 ± 0.1	38.7	−0.06 [−0.14, 0.02]					
Halbower *et al*.^26^	1.37 ± 0.41	0.94 ± 0.05	6.5	−0.03 [−0.05, −0.01]					
Kizilgoz *et al*.^34^	1.18 ± 0.92	1.25 ± 0.78	2.4	−0.07 [−0.63, 0.49]					
O’Donoghue *et al*.^32^	0.38 ± 0.04	0.41 ± 0.04	52.3	0.43 [0.10, 0.76]					
Pooled			100	−0.01 [−0.10, 0.08]	0.05	63	0.78	0.31	0.11
NAA/Cr in frontal lobe									
Alchanatis *et al*.^35^	1.58 ± 0.14	1.66 ± 0.17	23.5	−0.08 [−0.17, 0.01]					
Algin *et al*.^33^	1.61 ± 0.64	1.98 ± 0.2	22.4	−0.37 [−0.57, −0.17]					
Gharraf *et al*.^36^	1.33 ± 0.13	2.08 ± 0.15	23.3	−0.95 [−1.06, −0.84]					
Halbower *et al*.^26^	3.1 ± 1.7	2.7 ± 0.6	6.7	0.40 [−0.95, 1.75]					
O’Donoghue *et al*.^32^	1.59 ± 0.13	1.65 ± 0.18	23.5	−0.06 [−0.15, 0.03]					
Sarchielli *et al*.^29^	138.09 ± 6.9	147.09 ± 11	0.5	−9.00 [−14.69, −3.31]					
Pooled			100	−0.36 [−0.77, 0.06]	<0.01	97	0.09	0.45	0.15
NAA/Cho in frontal lobe									
Alchanatis *et al*.^35^	1.89 ± 0.43	1.78 ± 0.42	26.4	0.11 [−0.11, 0.33]					
Algin *et al*.^33^	1.48 ± 0.08	1.99 ± 0.36	28.0	−0.51 [−0.68, −0.34]					
Halbower *et al*.^26^	1.6 ± 0.4	2.2 ± 0.4	19.3	−0.60 [−1.04, −0.16]					
O’Donoghue *et al*.^32^	4.56 ± 0.41	4.92 ± 0.44	26.2	−0.36 [−0.59, −0.13]					
Pooled			100	−0.32 [−0.64, −0.01]	<0.01	86	0.05	0.31	0.50
Cho/Cr in frontal lobe									
Alchanatis *et al*.^35^	0.88 ± 0.19	0.99 ± 0.43	0.4	−0.11 [−0.31, 0.09]					
Algin *et al*.^33^	1.23 ± 0.14	1.03 ± 0.08	5.8	0.09 [0.04, 0.14]					
Gharraf *et al*.^36^	0.99 ± 0.08	1.17 ± 0.03	8.6	−0.18 [−0.22, −0.14]					
Halbower *et al*.^26^	1.9 ± 1.1	1.3 ± 0.3	0.0	0.60 [−0.25, 1.45]					
O’Donoghue *et al*.^32^	0.35 ± 0.02	0.33 ± 0.03	85.1	0.02 [0.01, 0.03]					
Sarchielli *et al*.^29^	96.99 ± 12.7	94.88 ± 5.19	0.0	2.11 [−3.90, 8.12]					
Pooled			100	−0.02 [−0.14, 0.09]	<0.01	94	0.68	0.99	0.35

Abbreviations: NAA, N-acetylaspartate; Cho, choline; Cr, creatine; OSA, Obstructive sleep apnea; MD, Mean difference; CI, Confidence Interval; *P*_Begg_, *p*-value for Begg’s test; *P*_Egger_, *p*-value for Egger test.

**Table 3:** Subgroup analysis of cerebral metabolites in the white matter and frontal cortex.

Study	OSA	Control	Weight%	WMD [95%CI]	*p*	I^2^%	*p*	*P*_Begg_	*P*_Egger_
NAA/Cr in frontal white matter									
Alchanatis *et al*.^35^	1.59 ± 0.16	1.74 ± 0.15	34.3	−0.15 [−0.26, −0.04]					
Algin *et al*.^33^	1.72 ± 0.8	2.15 ± 0.1	31.5	−0.43 [−0.76, −0.10]					
Gharraf *et al*.^36^	1.13 ± 0.13	2.08 ± 0.15	34.3	−0.95 [−1.06, −0.84]					
Pooled			100	−0.51 [−1.11, 0.09]	<0.01	98	0.09	0.30	0.07
Cho/Cr in frontal white matter									
Alchanatis *et al*.^35^	1.01 ± 0.15	1.19 ± 0.17	32.0	−0.18 [−0.30, −0.06]					
Algin *et al*.^33^	1.23 ± 0.1	1.05 ± 0.1	33.6	0.18 [0.10, 0.26]					
Gharraf *et al*.^36^	0.99 ± 0.08	1.17 ± 0.03	34.3	−0.18 [−0.22, −0.14]					
Pooled			100	−0.06 [−0.31, 0.19]	<0.01	97	0.64	0.99	0.72
NAA/Cr in frontal cortex									
Alchanatis *et al*.^35^	1.56 ± 0.12	1.58 ± 0.15	51.5	−0.02 [−0.13, 0.09]					
Algin *et al*.^33^	1.5 ± 0.4	1.8 ± 0.1	45.0	−0.30 [−0.47, −0.13]					
Halbower *et al*.^26^	3.1 ± 1.7	2.7 ± 0.6	3.4	0.40 [−0.95, 1.75]					
Pooled			100	−0.13 [−0.39, 0.13]	0.02	75	0.32	0.99	0.60
NAA/Cho in frontal cortex									
Alchanatis *et al*.^35^	2.18 ± 0.35	2.07 ± 0.36	34.1	0.11 [−0.15, 0.37]					
Algin *et al*.^33^	1.44 ± 0. 1	2.26 ± 0.3	34.9	−0.82 [−1.02, −0.62]					
Halbower *et al*.^26^	1.6 ± 0.4	2.2 ± 0.4	31.0	−0.60 [−1.04, −0.16]					
Pooled			100	−0.43 [−1.07, 0.20]	<0.01	94	0.18	0.30	0.26
Cho/Cr in frontal cortex									
Alchanatis *et al*.^35^	0.74 ± 0.13	0.78 ± 0.13	15.9	−0.04 [−0.14, 0.06]					
Algin *et al*.^33^	1 ± 0.04	1 ± 0.06	83.9	0.00 [−0.04, 0.04]					
Halbower *et al*.^26^	1.9 ± 1.1	1.3 ± 0.3	0.2	0.60 [−0.25, 1.45]					
Pooled			100	−0.01 [−0.04, 0.03]	0.29	20	0.80	0.30	0.22

NAA, N-acetylaspartate; Cho, choline; Cr, creatine; OSA, Obstructive sleep apnea; MD, Mean difference; CI, Confidence Interval; *P*_Begg_, *p*-value of Begg’s test; *P*_Egger_, *p*-value of Egger test.
