# Supplementary material for: Changes in cerebral metabolites in obstructive sleep apnea: a systemic review and meta-analysis
Source: Sci Rep. 2016 Jun 28;6:28712. doi: 10.1038/srep28712 (PMC4923864; doi:10.1038/srep28712)
Supplement: Supplementary Table 1 [file srep28712-s1.pdf]

Changes in cerebral metabolites in obstructive sleep apnea: a systemic review and meta-analysis

Yunyan Xia[1,2,3,*],MD, Yiqun Fu[1,2,3,*],MD, Huajun Xu[1,2,3],MD, Jian Guan[1,2,3], MD,PhD, Hongliang Yi[1,2,3], MD, PhD, and Shankai Yin[1,2,3], MD, PhD

1 Department of Otolaryngology Head and Neck Surgery& Center of Sleep Medicine, Shanghai Jiao Tong University Affiliated Sixth People's Hospital, Yishan Road 600, Shanghai, 200233, China.

2 Otolaryngological Institute of Shanghai Jiao Tong University, Yishan Road 600, Shanghai, 200233, China.

3 Clinical Research Center, Shanghai Jiao Tong University School of Medicine, South Chongqing Road 225, Shanghai,200020, China.

*these authors contributed equally to this paper.

Corresponding author: Huajun Xu, MD(E-mail:sunnydayxu2010@163.com), and Jian Guan, MD, PhD(E-mail: guanjian0606@sina.com)

Supplementary Table 1 Cerebral metabolites with different units in different studies in OSA and control group

Study	OSA	Control	*p*
**NAA in hippocampus**			
**Bartlett et al[31] ,2004**	9.94±6.68(relative to water resonance)	9.06±2.86(relative to water resonance)	1.00
**Kizilgoz et al[34] ,2013**	1.57±0.63(N/A)	2.36±0.42(N/A)	-
**Sharma et al[38] ,2010**	6.19±1.22(mmol/L)	6.13±0.93(mmol/L)	0.90
**Cr in hippocampus**			
**Bartlett et al[31] ,2004**	6.94±6.13(relative to water resonance)	9.62±3.79(relative to water resonance)	＜0.01
**Kizilgoz et al[34] ,2013**	0.8±0.13(N/A)	0.92±0.11(N/A)	-
**Sharma et al[38] ,2010**	4.78±1(mmol/L)	5.21±0.84(mmol/L)	0.29
**NAA in frontal lobe**			
**Alchanatis et al[35],2004**	7.04±0.97(mmol/kg wet weight)	7.64±0.89(mmol/kg wet weight)	-
**Gharraf et al[36],2014**	37±4.19(mmol/L)	47±1.70(mmol/L)	＜0.01
**Sharma et al[38],2010**	7.07±0.76(mmol/L)	7.03±1.06(mmol/L)	-
**Cho in frontal lobe**			
**Alchanatis et al[35],2004**	1.58±0.14(mmol/kg wet weight)	1.88±0.5(mmol/kg wet weight)	-
**Gharraf et al[36], 2014**	23.4±0.63(mmol/L)	27.5±1.51(mmol/L)	＜0.01
**Sharma et al[38],2010**	1.63±0.8(mmol/L)	1.38±0.4(mmol/L)	-
**NAA in frontal white matter**			
**Alchanatis et al[35],2004**	6.76±1.06(mmol/kg wet weight)	7.68±1.06(mmol/kg wet weight)	0.04
**Gharraf et al[36],2014**	37±4.19(mmol/L)	47±1.70(mmol/L)	＜0.01
**Sharma et al[38], 2010**	7.50±0.69(mmol/L)	7.57±2.07(mmol/L)	0.94
**Cho in frontal white matter**			
**Alchanatis et al[35], 2004**	1.75±0.33(mmol/kg wet weight)	2.18±0.44(mmol/kg wet weight)	0.02
**Gharraf et al[36],2014**	23.4±0.63(mmol/L)	27.5±1.51(mmol/L)	＜0.01
**Sharma et al[38],2010**	2.22±0.25(mmol/L)	1.87±0.21(mmol/L)	0.09

Abbreviations: NAA, N-acetylaspartate; Cho, choline; Cr, creatine; OSA, Obstructive sleep apnea; N/A, not afford.
