# Supplementary material for: Changes in cerebral metabolites in obstructive sleep apnea: a systemic review and meta-analysis
Source: Sci Rep. 2016 Jun 28;6:28712. doi: 10.1038/srep28712 (PMC4923864; doi:10.1038/srep28712)
Supplement: Supplementary Table 2 [file srep28712-s2.pdf]

Changes in cerebral metabolites in obstructive sleep apnea: a systemic review and meta-analysis

Yunyan Xia[1,2,3,*],MD, Yiqun Fu[1,2,3,*],MD, Huajun Xu[1,2,3],MD, Jian Guan[1,2,3], MD,PhD, Hongliang Yi[1,2,3], MD, PhD, and Shankai Yin[1,2,3], MD, PhD

1 Department of Otolaryngology Head and Neck Surgery& Center of Sleep Medicine, Shanghai Jiao Tong University Affiliated Sixth People's Hospital, Yishan Road 600, Shanghai, 200233, China.

2 Otolaryngological Institute of Shanghai Jiao Tong University, Yishan Road 600, Shanghai, 200233, China.

3 Clinical Research Center, Shanghai Jiao Tong University School of Medicine, South Chongqing Road 225, Shanghai,200020, China.

*these authors contributed equally to this paper.

Corresponding author: Huajun Xu, MD(E-mail:sunnydayxu2010@163.com), and Jian Guan, MD, PhD(E-mail: guanjian0606@sina.com)

Supplementary Table 2 Sensitivity analysis of the results of changes in cerebral metabolite

Study	OSA	Control	Weight%	MD [95%CI]	*p*	I^2%	*p*	P_{Begg}	P_{Egge}
**NAA/Cho in hippocampus(with exclusion of the study of Halbower et al)**									
**Algin et al[33],2012**	1.07±0.1	1.11±0.2	74.5	–0.04 [–0.18, 0.10]					
**Kizilgoz et al[34],2013**	2.06±0.99	2.25±0.67	5.2	–0.19 [–0.71, 0.33]					
**O'Donoghue et al[32],2012**	4.15±0.52	4.04±0.45	20.3	0.11 [–0.15, 0.37]					
**Pooled**			100	–0.12 [–0.36, 0.10]	0.49	0	0.77	0.30	0.09
**Cho/Cr in hippocampus(with exclusion of the study of Halbower et al)**									
**Algin et al[33],2012**	1.09±0.1	1.15±0.1	0.1	–0.06 [–0.14, 0.02]					
**Kizilgoz et al[34],2013**	1.18±0.92	1.25±0.78	7.4	–0.07 [–0.63, 0.49]					
**O'Donoghue et al[32],2012**	0.38±0.04	0.41±0.04	92.4	0.43 [0.10, 0.76]					
**Pooled**			100	–0.01 [–0.10, -0.01]	0.75	0	<0.01	1.00	0.68
**NAA/Cho in frontal lobe(with exclusion of the study of Alchanatis et al)**									
**Algin et al[33],2012**	1.48±0.08	1.99±0.36	59.8	–0.51 [–0.68, –0.34]					
**Halbower et al[26],2006**	1.6±0.4	2.2±0.4	8.9	–0.60 [–1.04, –0.16]					
**O'Donoghue et al[32],2012**	4.56±0.41	4.92±0.44	31.3	–0.36 [–0.59, –0.13]					
**Pooled**			100	–0.32 [–0.64, –0.34]	0.49	0	<0.01	1.00	0.82
**NAA/Cho in frontal lobe(with exclusion of the study of Algin et al)**									
**Alchanatis et al[35],2004**	1.89±0.43	1.78±0.42	36.3	0.11 [–0.11, 0.33]					
**Halbower et al[26],2006**	1.6±0.4	2.2±0.4	27.6	–0.60 [–1.04, –0.16]					
**O'Donoghue et al[32],2012**	4.56±0.41	4.92±0.44	36.0	–0.36 [–0.59, –0.13]					
**Pooled**			100	–0.26 [–0.66, 0.15]	<0.0	84	0.21	1.00	0.57
**NAA/Cho in frontal lobe(with exclusion of the study of Halbower et al)**									
**Alchanatis et al[35],2004**	1.89±0.43	1.78±0.42	32.8	0.11 [–0.11, 0.33]					
**Algin et al[33],2012**	1.48±0.08	1.99±0.36	34.6	–0.51 [–0.68, –0.34]					
**O'Donoghue et al[32],2012**	4.56±0.41	4.92±0.44	32.5	–0.36 [–0.59, –0.13]					
**Pooled**			100	–0.26 [–0.63, 0.11]	<0.0	90	0.17	0.30	0.14
**NAA/Cho in frontal lobe(with exclusion of the study of O'Donoghue et al)**									
**Alchanatis et al[35],2004**	1.89±0.43	1.78±0.42	26.4	0.11 [–0.11, 0.33]					
**Algin et al[33],2012**	1.48±0.08	1.99±0.36	28.0	–0.51 [–0.68, –0.34]					
**Halbower et al[26],2006**	1.6±0.4	2.2±0.4	19.3	–0.60 [–1.04, –0.16]					
**Pooled**			100	–0.32 [–0.78, 0.15]	<0.0	90	0.18	1.00	0.82

Abbreviations: NAA, N-acetylaspartate; Cho, choline;Cr, creatine; OSA, Obstructive sleep apnea; MD, Mean difference; CI, Confidence Interval; P_{Begg}, *p*-value of Begg's test; P_{Egger}, *p*-value of Egger test
